# Supplementary material for: One-Pot Synthesis of Dual Color-Emitting CDs: Numerical and Experimental Optimization towards White LEDs
Source: Nanomaterials (Basel). 2023 Jan 17;13(3):374. doi: 10.3390/nano13030374 (PMC9920914; doi:10.3390/nano13030374)
Supplement: Supplementary file 1 [file nanomaterials-13-00374-s001.zip › nanomaterials-2120782-supplementary.pdf]

Article

One-Pot Synthesis of Dual Color-Emitting CDs: Numerical and Experimental Optimization towards White LEDs

Gianluca Minervini [1,2†], Antonino Madonia [2†], Annamaria Panniello [2], Elisabetta Fanizza [3,2], Maria Lucia Curri [3,2], Marinella Striccoli [2*]

[1] Department of Electrical and Information Engineering, Polytechnic of Bari, Via E. Orabona 4, Bari, 70126, Italy; gianluca.minervini@poliba.it

[2] CNR-IPCF Bari Division, c/o Chemistry Department, University of Bari "Aldo Moro", Via Orabona 4, Bari, 70126, Italy; a.madonia@ba.ipcf.cnr.it (A.M.); a.panniello@ba.ipcf.cnr.it (A.P.);

[3] Department of Chemistry, University of Bari "Aldo Moro", Via Orabona 4, Bari, 70126, Italy; elisabetta.fanizza@uniba.it (E.F.); marialucia.curri@uniba.it (M.L.C.);

* Correspondence: m.striccoli@ba.ipcf.cnr.it;

† These authors contributed equally to this work

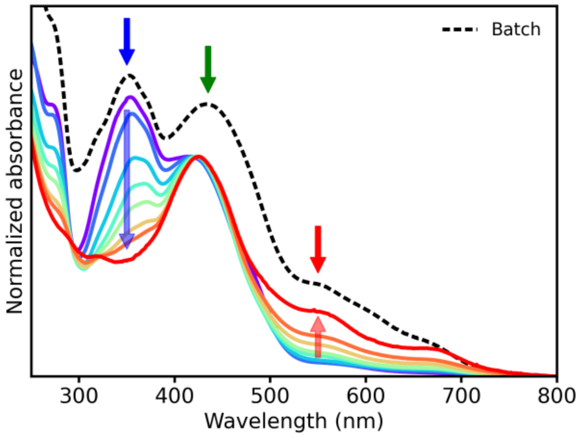

Figure S1. Absorption spectra of the CDs crude synthetic batch (dashed black line) and of the supernatants discarded at each purification step (color lines). It is possible to follow the progressive disappearing of the bands responsible for the blue and green emitting by-products alongside the appearance of the CDs' red emission band, as indicated by the arrows.

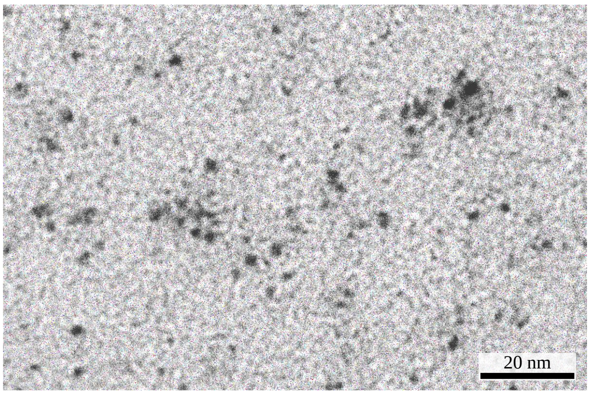

Figure S2. TEM image of CDs; some aggregates of several nanoparticles sized around 10 nm in diameter can be observed. Based on the ratio between the average areas of aggregates and nanoparticles, the former appear composed by up to 20 CDs.

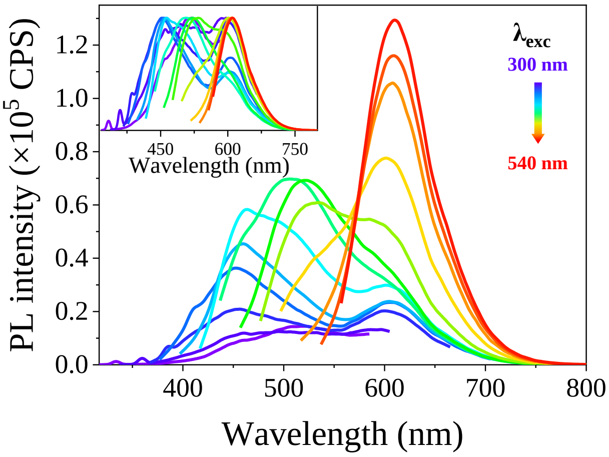

Figure S3. PL spectra and normalized PL spectra (inset) of CDs aqueous dispersions recorded exciting at λ_{exc} in the range 300 – 540 nm at an interval of 20 nm. The excitation dependent emission of CDs (evidenced in the inset) results from the overlap of three different emission bands. The narrow peak with low intensity at very short wavelengths appearing at a fixed shift (of around 30–40 nm, corresponding to 3400-3600 cm^{-1}) from the excitation wavelength can be interpreted as a spurious Raman signal, ascribable to the O–H symmetric stretching vibration of the water used as solvent [91].

TR decay fit and analysis

A bi-exponential decay was needed to fit the time traces: under irradiation at 485 nm it is possible to excite both the green and red band at the same time, each one decaying with its own lifetime.
The model used for the least-squares fitting procedure is shown in (S1 and S2):

$$y(t) = f * \mathrm{IRF}(t) \quad \text{(S1)}$$

$$f(t) = A_1 e^{\frac{-t-t_0}{\tau_1}} + A_2 e^{\frac{-t-t_0}{\tau_2}} \quad \text{(S2)}$$

The model function $y(t)$ is the convolution between a bi-exponential decay ($f(t)$, S2) and the instrument response function (IRF(t), S1); the decay trace of the 485 nm excitation laser passing through a scattering solution was used as instrument response function. In the bi-exponential decay, A_i are the amplitudes of the two exponential components, t_0 is the time at which the emission decay starts and τ_i are the lifetimes of the two decay components. Fit results and relative residuals are shown in Figure S3 and Table S1:

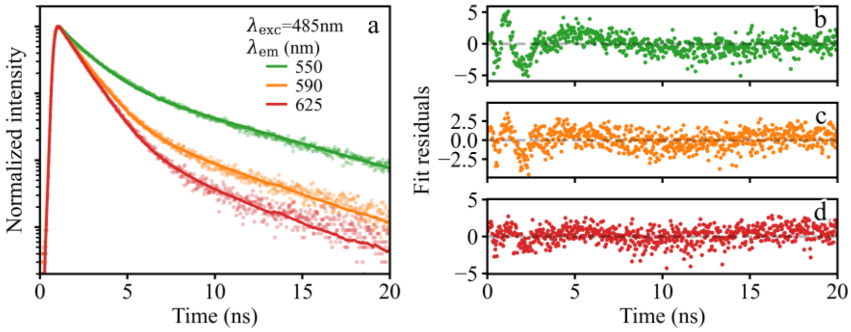

Figure S4. fit results (a) and relative residuals (b, c, d).

Table S1. Best-fitting parameters obtained from the least-squares minimization procedure performed on the time-resolved emission traces of CDs (λ_{exc} = 485 nm)

λ_{em} (nm)	A_1 rel. (%)	τ_1 (ns)	A_2 rel.	τ_2 (ns)
550	54.5 ± 0.2	1.26 ± 0.10	45.5 ± 0.2	5.79 ± 0.10
590	83.13 ± 0.14	1.03 ± 0.10	16.87 ± 0.14	4.65 ± 0.11
625	90.76 ± 0.12	0.96 ± 0.10	9.24 ± 0.12	3.96 ± 0.11

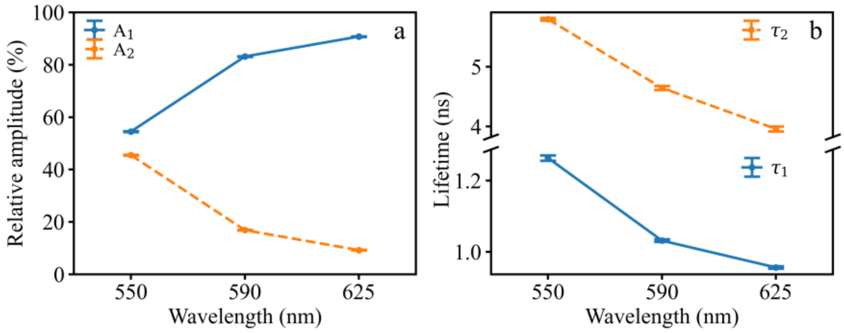

Figure S5. Comparison between the relative amplitudes (a) and lifetimes (b) obtained from the fit.

Equation (2): choice of normalization conditions and definitions of relative quantities

The spectral densities of irradiance (in W·cm-2·nm-1) for the blue and green/red components are called $\Phi^{B}(\lambda)$and $\Phi^{GR}(\lambda)$. The spectral density of the total irradiance ($\Phi(\lambda)$, also in W·cm-2·nm-1) is defined as:

$$\Phi(\lambda) = \Phi^{B}(\lambda) + \Phi^{GR}(\lambda) \tag{S3}$$

Since colorimetric calculations (such as those of CIE coordinates, CCT and CRI) are made using normalized spectral irradiance quantities [86], we can refer to a dimensionless relative spectral density of irradiance (or relative color stimulus function), called $\phi(\lambda)$.

$\phi(\lambda)$ will be given by the physically measured quantity $\Phi(\lambda)$, divided by a normalization factor (k):

$$\phi(\lambda) = \frac{\Phi(\lambda)}{k} \tag{S4}$$

The normalization condition that defines k (and hence $\phi(\lambda)$) can be set according to specific convenience for the case. Herein, we choose to define k as the integrated area of $\Phi(\lambda)$, called A_{TOT} (in W·cm^{-2}) multiplied by 1 nm (to ensure that $\phi(\lambda)$ is dimensionless):

$$k = A_{TOT} \cdot 1nm \tag{S5}$$

In this way, called A^{GR} and A^{B} the integrated areas of $\Phi^{GR}(\lambda)$ and $\Phi^{B}(\lambda)$, the following relations hold:

$$k = (A^{GR} + A^{B}) \cdot 1nm \tag{S6}$$

$$\Phi^{GR}(\lambda) = A^{GR}\phi^{GR}(\lambda) \cdot 1nm \quad \text{(S7)}$$

$$\Phi^{B}(\lambda) = A^{B}\phi^{B}(\lambda) \cdot 1nm \quad \text{(S8)}$$

by substituting Equations (S6, S7 and S8) and Equation (S4) into Equation (S3) it is possible to obtain Equation (2) and Equation (3-4).

Derivation of Equations (5-6) and underlying assumptions

We consider as primary source a parallel light beam with an in-plane irradiance I_0^S (W·cm^{-2}), characterized by a monochromatic wavelength in the blue that is incident on the surface of a nanocomposite slab. While propagating, the primary blue beam is attenuated, meanwhile part of the absorbed radiation is converted into light at longer wavelengths, forming the green and red emission bands.

Neglecting reflections and scattering at the surface of the nanocomposite, as well as assuming that the generated spectrum, composed of the attenuated primary blue light and PL emitted light are collected at a 360° angle and within a spectral rage covering all the transmitted and emitted wavelengths, the irradiance of the attenuated transmitted blue beam (I^S) and of the PL generated in the nanocomposite (I^{PL}) can be easily calculated with the equations:

$$I^S = I_0^S\, e^{-\alpha_B d} \quad \text{(S9)}$$

$$I^{PL} = QY\left(I_0^S - I^S\right) \quad \text{(S10)}$$

Where αB, d and QY are the absorption coefficient at the incident wavelength, nanocomposite slab thickness, and QY of fluorophores in the nanocomposite, measured exciting at the incident light wavelength.

Since the incident radiation is monochromatic, and if the emission spectrum is collected over all the emitted wavelengths, both attenuated incident and emitted irradiances are equal to the areas of their spectral distribution. In general, if the spectra are recorded over a sufficiently broad range, we can estimate the incident and emitted irradiances from the areas of their spectral distributions:

$$I^S \approx A^B \quad \text{(S11)}$$

$$I^{PL} \approx A^{GR} \quad \text{(S12)}$$

Substituting Equations (S11 and S12) in Equation (3) we obtain:

$$f_m \approx \frac{I^{PL}}{I^S + I^{PL}} \quad \text{(S13)}$$

and substituting Equations (S9 and S10) into Equation (S13) we obtain Equations (5-6).

Table S2. Best achievable white colorimetric properties calculated for CDs, compared with the colorimetric properties of illuminant D65.

Sample	$\boldsymbol{f_{baw}}$	$\boldsymbol{(x, y)_{baw}}$	$\boldsymbol{CCT_{baw}}$ (K)	$\boldsymbol{CRI_{baw}}$
CDs	0.64	(0.31, 0.33)	6540	82
D65	/	(0.31272, 0.32903)	6504	100

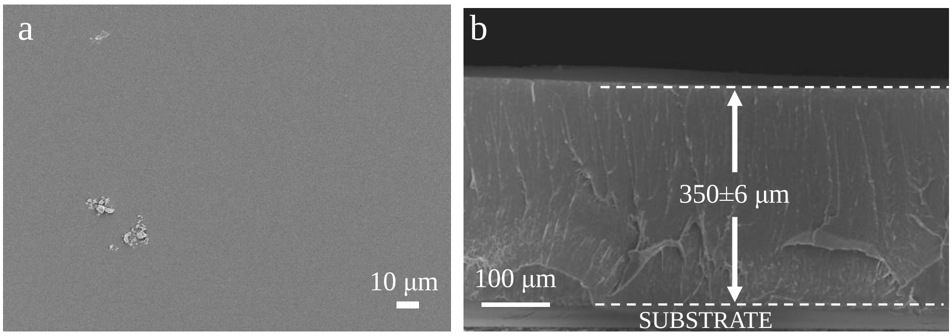

Figure S6. (a) In-plane and (b) cross-section SEM micrographs of the drop-casted nanocomposite film DC-B.

Table S3. Thicknesses of drop-casted films DC-B and DC-E measured form cross-section SEM micrographs.

Sample	dSEM (μm)
DC-B	350±6
DC-E	126±2

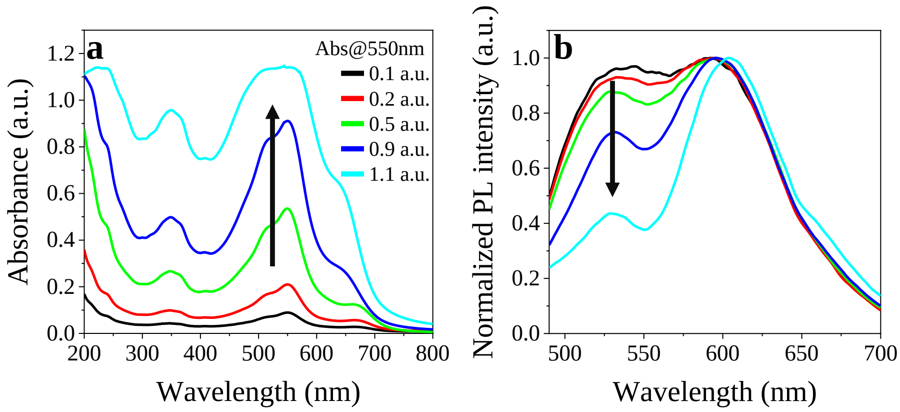

Figure S7. (a) UV-vis absorption and (b) normalized PL spectra (λ_{exc} = 465 nm) of CDs aqueous dispersions at increasing concentrations. Differences in the PL spectral shape are attributed to photon re-absorption in the more concentrated CDs dispersions.

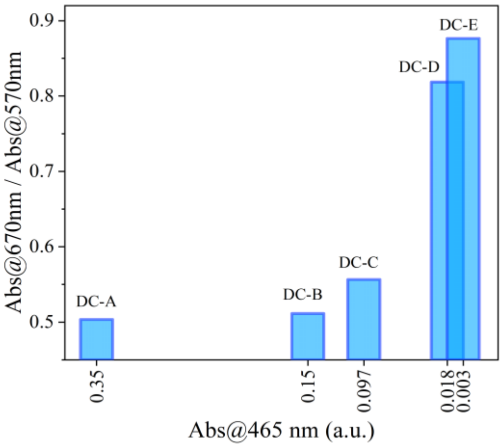

Figure S8. Ratio of the absorbance at 670 nm and at 570 nm in the absorption spectra of nanocomposite films prepared by drop-casting as a function of the absorbance at 465 nm going from sample DC-A to DC-E.

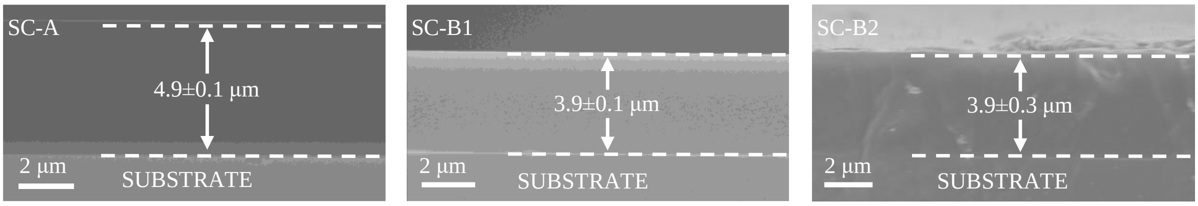

Figure S9. Cross-section SEM micrographs of spin-coated nanocomposite films SC-A, SC-B1 and SC-B2.

Estimate of film thickness from interference fringes in UV-Vis absorption spectra of nanocomposite films prepared by spin-coating

Interference fringes in the transparency region of the UV-Vis Absorption spectrum (800 – 900 nm) can be used to estimate the thickness of film, with the formula [92]:

$$d = \frac{M\lambda_1\lambda_2}{2n(\lambda_2 - \lambda_1)} \quad \text{(S14)}$$

Where: λ_1, λ_2 are the wavelengths of two selected extrema (maxima or minima), M is the number of oscillations between the two selected extrema (maxima or minima) and n is the refractive index of the film. For our calculation, n is assumed to be equal of that of PVA at 800 nm, i.e. n = 1.48.

Calculation of deviations of experimentally measured CIE chromaticity coordinates, CCT and CRI from colorimetric properties of CIE illuminant D65

The deviation Δ CIE of the experimentally measured chromaticity coordinates (called x_{exp} and y_{exp}) from the chromaticity coordinates of CIE illuminant D65 (called x_{D65} and y_{D65}) was calculated as:

$$\Delta\, CIE = \left(x_{exp} - x_{D65}\right)^2 + \left(y_{exp} - y_{D65}\right)^2 \quad \text{(S15)}$$

Percentage deviation of CCT (ΔCCT%) and deviation of CRI (ΔCRI) are respectively calculated with the formulas:

$$\Delta CCT\% = \frac{\left|CCT_{exp} - CCT_{D65}\right|}{CCT_{D65}} \times 100 \quad \text{(S16)}$$

$$\Delta CRI = \mathrm{CRI_{D65}} - CRI_{exp} \quad \text{(S17)}$$

Where CCT_{exp} and CRI_{exp} are the experimentally measured CCT and CRI, CCT_{D65} = 6500 K and CRI_{D65} = 100 are CCT and CRI of standard illuminant D65.

Reference list:

[86] Oleari, C. *Standard Colorimetry: Definitions, Algorithms, and Software*, John Wiley & Sons, Inc: Chichester, UK, 2016; ISBN 978-1-118-89445-3.

[91] Baschenko, S.M.; Marchenko, L.S. On Raman spectra of water, its structure and dependence on temperature. *Semicond. Phys. Quantum Electron. Optoelectron.* **2011**, *14*, *77–79*. https://doi.org/10.15407/spqeo14.01.077.

[92] Manifacier, J.C.; Gasiot, J.; Fillard, J.P. A Simple Method for the Determination of the Optical Constants n, h and the Thickness of a Weakly Absorbing Thin Film. *J. Phys. E: Sci. Instrum.* **1976**, *9*, 1002–1004.
